# Regulation of magnesium ion transport in *Escherichia coli*: insights into the role of the 5’ upstream region in *corA* expression

**DOI:** 10.1080/15476286.2024.2421665

**Published:** 2024-11-08

**Authors:** A.-S. Vézina Bédard, A. Michaud, F. Quenette, N. Singh, F. de Lemos Martins, J.T. Wade, M. Guillier, D.A. Lafontaine

**Affiliations:** a Department of Biology, Faculty of Science, RNA Group, Université de Sherbrooke, Sherbrooke, QC, Canada; b Expression Génétique Microbienne, UMR8261 CNRS, Université Paris Cité, Institut de Biologie Physico-Chimique, Paris, France; c Wadsworth Center, New York State Department of Health, Albany, NY, USA; d Department of Biomedical Sciences, University at Albany, Albany, NY, USA

**Keywords:** Genetic regulation, leader region, magnesium homeostasis, regulatory RNA, corA

## Abstract

In *Escherichia coli*, transport of magnesium ions across the cellular membrane relies on MgtA and CorA transporters. While the expression of *mgtA* is controlled by the two-component system PhoQ/PhoP and 5’ upstream region elements, *corA* expression is considered to be constitutive and not to depend on cellular factors. Importantly, the 5’ upstream region of *corA* is predicted to fold into structures highly similar to the magnesium-sensing *mgtA* 5‘ upstream region. Here using biochemical and genetic assays, we show that the intracellular concentration of magnesium ions affects *corA* expression. Similarly to *mgtA*, we find that the effect of magnesium ions on *corA* expression is mediated by the 5’ upstream region. We demonstrate that the RNA structure is important for regulation and that the Rho transcription factor is involved in the modulation of transcription termination. Consistent with previous studies, we find that translation of *corL*, a short ORF located within the 5’ upstream region, is important for *corA* regulation. Our data indicate that the efficiency of *corL* translation is inversely proportional to *corA* expression, similar to what has been described for *mgtA* and *corA* in *Salmonella enterica*. Using a novel assay to control the import of magnesium ions, we show that while the expression of *mgtA* is regulated by both extra- and intracellular magnesium ions, *corA* is regulated by variations in intracellular magnesium ions. Our results support a model in which the expression of *corA* is regulated by the 5’ upstream region that senses variations of intracellular magnesium ions.

## Introduction

In bacteria, the intracellular concentrations of magnesium ions is important for enzymatic activity and for maintaining membrane integrity [1,2]. The concentration of free magnesium ions can reach 0.3 ~ 1.0 mM internally, while the total Mg^2+^ concentration in Gram-negative bacteria like *Escherichia coli* can reach as high as 100 mM [3]. To preserve high magnesium ion concentrations in the cell, bacteria possess several specific transporters. While *E. coli* relies on the two transporters CorA and MgtA, *Salmonella enterica* uses CorA, MgtA and MgtB [3]. Transcription of *mgtA* is regulated by the two-component system PhoQ/PhoP, which consists of the PhoQ sensor kinase and the PhoP response regulator [4,5]. This system is triggered at low intracellular magnesium concentrations to induce promoter activity [3,6], thus leading to a rise in intracellular Mg^2+^ levels.

**Figure 1.**
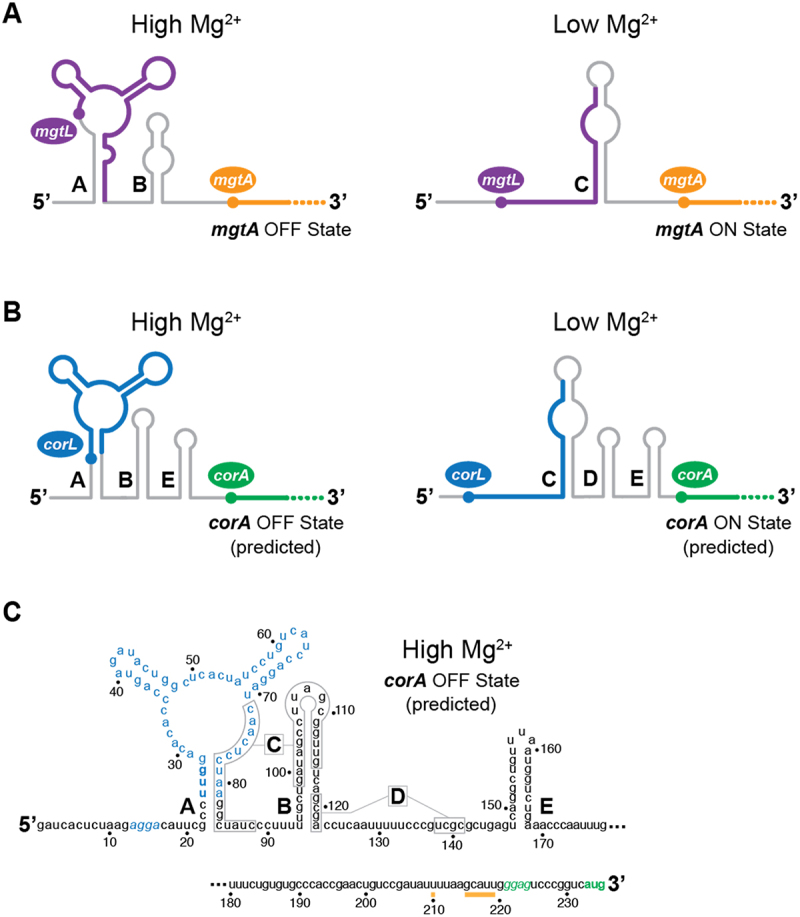
The *mgtA* and *corA* 5’ upstream regions of *Escherichia coli*.

Expression of *mgtA* is also modulated by control elements located in the 5’ upstream region (UR) [7]. For instance, high concentrations of magnesium ions downregulate the expression of *mgtA* [7], which is mediated by Rho-dependent transcription termination [8,9]. This regulatory mechanism was reported to primarily depend on an upstream open reading frame (uORF), *mgtL*, that is located in *mgtA* 5’ UR [10,11]. The presence of prolines and acidic residues in the uORF was found to be important for *mgtA* regulation [10,12,13]. The most recent studies show that, at low magnesium concentration, inefficient *mgtL* translation elongation – caused by the presence of proline or acidic residues – leads to high levels of *mgtA* expression. Reciprocally, an increase in intracellular Mg^2+^ allows efficient *mgtL* translation elongation, leading to *mgtA* repression [12,13]. Structural probing showed that the *mgtA* 5’ UR adopts two mutually exclusive structures at high and low Mg^2+^ concentrations [7,11]. At high Mg^2+^ concentration, the RNA structure is predicted to contain stem-loops A and B (Figure 1(A)). However, at low Mg^2+^ concentration, the RNA is expected to contain stem-loop C (Figure 1(A)). It is still not fully understood whether the sensing of Mg^2+^ ions is performed through direct RNA binding or the efficiency of *mgtL* translation elongation. Nevertheless, all available data are consistent with changes in magnesium concentrations modulating the expression of *mgtA* [7,10–14].

(A and B) The structures of *mgtA* (A) and *corA* (B) 5’ upstream regions are indicated for High Mg^2+^ (OFF state) and Low Mg^2+^ (ON state). Bold lines represent the uORF *mgtL* and *corL*. The capital letters indicate the nomenclature of the stems (A to E). The ON and OFF states are associated with active and inactive gene expression, respectively. While the structures of the *mgtA* 5’ upstream region have been previously associated to the low and high magnesium concentrations, the structures of the *corA* 5’ upstream region are predicted to be adopted in each condition. (C) Secondary structure of the *corA* 5’ upstream region. The structure is represented in the OFF state conformation that is predicted to be adopted at high magnesium concentrations. The 5’ upstream region exhibits a three-way junction structure (positions 22 to 84) and two stem-loops (B and E). The regions involved in the alternative stem-loops C and D are indicated. Rho termination sites are represented by orange lines. Nucleotides in blue and green represent *corL* and *corA* sequences, respectively, which include the Shine-Dalgarno (italics) and start codon (bold).

It was previously reported that the *S. enterica corA* 5’ UR adopts two mutually exclusive structures (Figure 1(B)) [15] that are highly similar to those observed in *mgtA* (Figure 1(A)). For instance, the OFF state is characterized by stems A and B, the latter being flanked by pyrimidine-rich regions constituting a *rut* site [15]. The ON state exhibits the C and D stems that sequester the *rut* sites, and thus permits efficient transcription elongation. Both the OFF and ON states contain the stem E, which has been shown to prevent Rho-dependent termination before *corA* coding region [16]. Similarly to *mgtA*, a uORF (*corL*) is also present within the stem-loop A (Figures 1(B,C)) [15,17]. Expression of *corL* was determined to be inversely related to that of *corA* [15]. Because of these characteristics shared by *mgtA* and *corA*, we reasoned that the expression of *corA* might be regulated by changes in Mg^2+^ cellular concentrations. Importantly, despite the *corA* 5' UR exhibiting key structural elements of *mgtA* 5' UR, no direct evidence of magnesium-dependent regulation has yet been reported [18,19].

Here, we demonstrate that in *E. coli*, *corA* expression is regulated by changes in magnesium ion concentrations. In contrast to *mgtA*, we demonstrate that *corA* expression is not modulated at the promoter level but rather solely depends on 5' UR regulatory elements. As a result, our data are consistent with a model in which *corA* expression is only responsive to changes in intracellular magnesium ion concentrations. Our results indicate that *corL* expression is inversely proportional to that of *corA*, suggesting a similar regulatory mechanism to that proposed for *S. enterica corA* [15]. Together, our study shows that intracellular variations of magnesium ions modulate *corA* expression through regulatory elements in the 5' UR.

## Results

### *corA* expression is regulated by magnesium ion concentrations in *Escherichia coli*

It was previously reported that *corA* is a housekeeping gene because its expression was not found to be regulated under various cellular stresses [18,19]. However, the modulation of *corA* mRNA levels by Rho and RNase III activities suggest that *corA* expression could be regulated under specific cellular conditions [15,20]. Importantly, in contrast to *mgtA*, none of these studies has established whether changes in intracellular magnesium ion concentrations affect *corA* expression, even though published data suggested a decrease in CorA protein levels in response to high magnesium ions in stationary phase [21].

**Figure 2.**
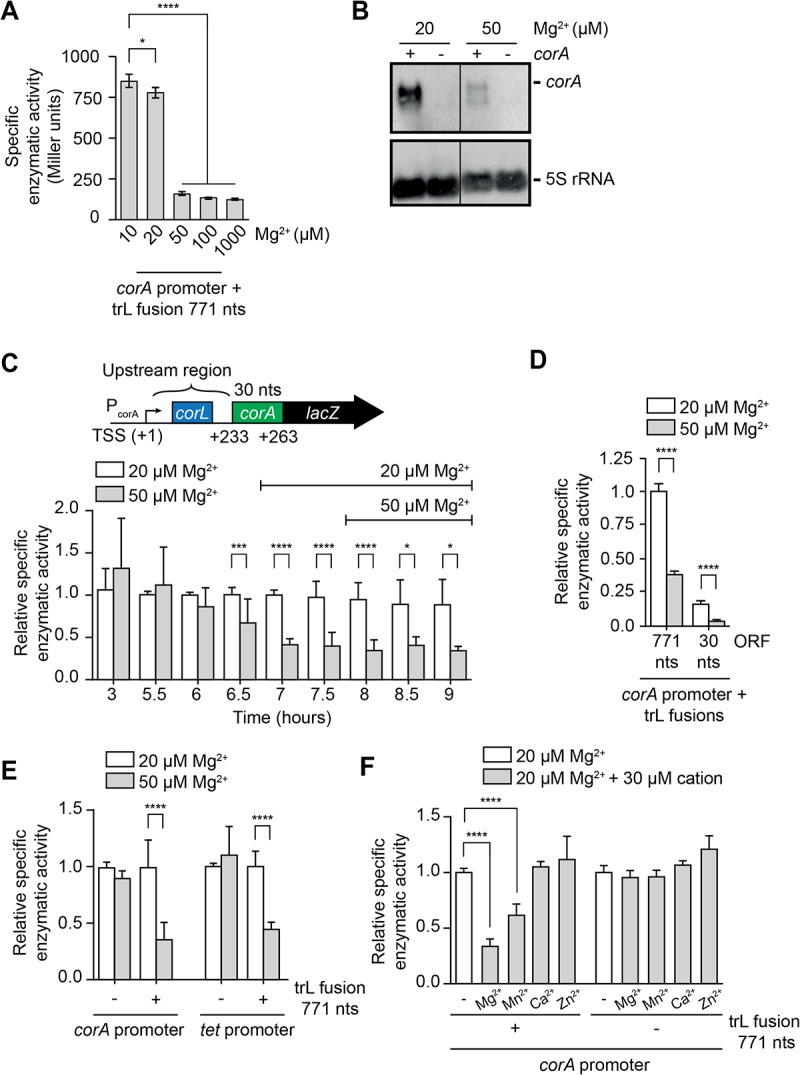
*corA* regulation relies on the 5' upstream region and is affected by magnesium ions.

To address the regulation of *corA*, we followed the expression of a *corA-lacZ* translational fusion at different magnesium concentrations in cells grown to an optical density of 1.5. This fusion is expressed from the *corA* promoter and contains 771 nts of *corA* coding sequence (*i.e*. 257 aa) (Figure 2(A) and see Supplementary Figure S1A for the P_corA_-*UR*_*WT*_-*corA*_*771*_-*lacZ* construct), consistent with a previously described well-expressed fusion [22]. While the expression of *corA* was efficient at 10 µM and 20 µM, it was much lower at 50 µM MgSO_4_ and above (Figure 2(A)), similarly to what reported for *mgtA* regulation [7]. As the strongest difference was observed between 20 µM and 50 µM MgSO_4_, these growth conditions were kept throughout the study. We then performed Northern blot experiments using a probe targeting the *corA* open reading frame (ORF). In these experiments, cells were grown in the presence of 20 µM or 50 µM magnesium and total RNA was extracted after cultures reached an OD_600_ of ~1.5. When supplemented with 20 µM MgSO_4_, strong expression of *corA* was observed (Figure 2(B)). No such signal was detected when using a *∆corA* strain (Figure 2(B)), indicating that the signal is specific to *corA*. However, when cells were grown using 50 µM MgSO_4_, *corA* expression was strongly decreased, confirming that variations of magnesium ions regulate *corA* mRNA levels.

(A) β-galactosidase assays using the P_corA_-*UR*_*WT*_-*corA*_*771*_-*lacZ* translational fusion at various magnesium ion concentrations. The average and the standard deviations are shown. The strain used in this experiment is FM02. (B) Northern blot analysis of *corA* mRNA level in *E. coli* strains with 20 or 50 µM MgSO_4_. Experiments were performed in wild-type (+ *corA*, DJ624 strain) or in a ∆*corA* mutant (- *corA*, MG1597 strain). 5S rRNA was used as a loading control. (C) β-galactosidase assays using the P_corA_-*UR*_*WT*_-*corA*_*30*_-*lacZ* translational fusion (strain MG2314) at various time point of the bacterial growth in a minimal media with 20 or 50 µM MgSO_4_. The average and the standard deviations are shown. The data have been normalized to that obtained at 5.5 h using 20 µM Mg^2+^. The horizontal bars indicate the time points corresponding to the stationary growth phase. A schematic representation of the construct is shown above the histograms. The transcription start site (TSS) and the relative positions of *corA* coding region are indicated on the construct. (D) β-galactosidase assays using the P_corA_-*UR*_*WT*_-*corA*_*30*_-*lacZ* (30 nts, strain MG2314) and P_corA_-*UR*_*WT*_-*corA*_*771*_-*lacZ* (771 nts, strain FM02) constructs performed in a minimal media with 20 or 50 µM MgSO_4_. The data obtained for the 30 nts construct have been normalized to that of the 771 nts construct obtained at 20 µM Mg^2+^. The average and the standard deviations are shown. (E) β-galactosidase assays using translational fusions expressed from the *corA* or *tet* promoter without (-) or with (+) the translational fusion containing 771 nts. Experiments were performed in minimal media with 20 or 50 µM MgSO_4_. The fusions expressed from the *corA* promoter consist of P_corA_-*lacZ* (- trL fusion 771 nts, strain MG2297) and P_corA_-*UR*_*WT*_-*corA*_*771*_-*lacZ* (+ trL fusion 771 nts, strain FM02) and those expressed from the *tet* promoter consist of P_tet_-*lacZ* (- trL fusion 771 nts, strain MG2316) and P_tet_-*UR*_*WT*_-*corA*_*771*_-*lacZ* (+ trL fusion 771 nts, strain FM06). Values were normalized to the activity obtained in 20 µM MgSO_4_. The average and the standard deviations are shown. (F) β-galactosidase assays of the P_corA_-*lacZ* (- trL fusion 771 nts, strain MG2297) and P_corA_-*UR*_*WT*_-*corA*_*771*_-*lacZ* (+ trL fusion 771 nts, strain FM02) fusions in minimal media with 20 µM MgSO_4_ and 30 µM of different cations. Values were normalized to the activity obtained in 20 µM MgSO_4_. The average and the standard deviations are shown.

To characterize in more detail the mechanism of *corA* regulation, we created different versions of the *corA-lacZ* fusion. A translational fusion was made that carried the *corA* promoter, 5' UR and the first 30 nts of *corA* ORF (P_corA_-*UR*_*WT*_-*corA*_*30*_-*lacZ*). The second construct consisted in a promoter fusion between P_corA_ and *lacZ* (P_corA_-*lacZ*). We monitored the enzymatic activity of each fusion at different time points along the bacterial growth to determine if these fusions were still regulated by magnesium ions and, if so, at what time points. We observed that changes in magnesium concentrations (20 µM vs 50 µM) affected the expression of the translational *corA-lacZ* fusion only after 7 h of bacterial growth or more (Figure 2(C)). This time point corresponds to the onset of the bacterial stationary phase (Supplementary Figure S2A), in agreement with CorA protein levels being affected by magnesium ions in stationary phase, but not in exponential phase [21]. In contrast, the promoter fusion did not respond to magnesium ions under these conditions, regardless of the stage of growth (Supplementary Figure S2B).

At last, we compared the fusions containing 30 or 771 nts of *corA* ORF at 7 h of growth in media containing 20 µM or 50 µM magnesium ions. Expression of both fusions was similarly reduced at 50 µM MgSO_4_ when compared to 20 µM, but the longer construct exhibited ~ 6.1-fold more expression when compared to the 30 nts construct (Figure 2(D)). Because of this more robust expression, later experiments employed the 771 nts construct.

### The regulation of *corA* is independent of the promoter region

The above results strongly suggested that the *corA* promoter is not regulated by changes in magnesium ion concentrations (Supplementary Figure S2B). To confirm this, we next used constructs in which the *corA* promoter was replaced by the tetracycline promoter, and in which the 5' UR was either preserved or removed (see Supplementary Figure S1B for constructs). When monitoring the expression of fusions carrying the *tet* promoter, we found that while no regulation was observed without the 5' UR (Figure 2(E), *tet* promoter, ‘− trL fusion 771 nts’), the Mg^2+^-dependent regulation was retained in the presence of the 5' UR (Figure 2(E), *tet* promoter, ‘+ trL fusion 771 nts’). Very similar results were obtained when using constructs containing the *corA* promoter (Figure 2(E)). Clearly, our results show that *corA* expression is modulated by variations in magnesium ion concentrations in a promoter-independent manner, and Mg^2+^-dependent regulation requires the presence of the 5' UR.

We next assessed *corA* gene regulation when growing cells using different metal ions. To do so, we performed β-galactosidase assays in a background of 20 µM magnesium ions and supplemented the media with 30 µM of the tested ions (Figure 2(F)). Control experiments confirmed that the type of magnesium salt did not affect the regulation (e.g. MgCl_2_ vs MgSO_4_) (Supplementary Figure S3). When testing various cations, we found that manganese ions reduced (~1.6-fold) *corA* expression, which yielded a smaller effect than with magnesium ions (Figure 2(F)). This result suggest that in addition to magnesium ions, the *corA* 5' UR responds to manganese ions, which is in contrast to the *mgtA* 5' UR [7]. No effect on *corA* regulation was observed when using calcium or zinc (Figure 2(F)), which is similar to *mgtA* regulation [7]. We also attempted to assess *corA* regulation using Co^2+^ and Ni^2+^ cations but were not able to grow cells in such conditions (data not shown). Importantly, no decrease in gene expression was obtained when using a construct containing only the promoter region (Figure 2(F)). These results indicate that regulation of *corA* at the promoter level does not respond to any of the tested ions.

### *corA* is regulated at the transcriptional level by Rho-dependent termination

**Figure 3.**
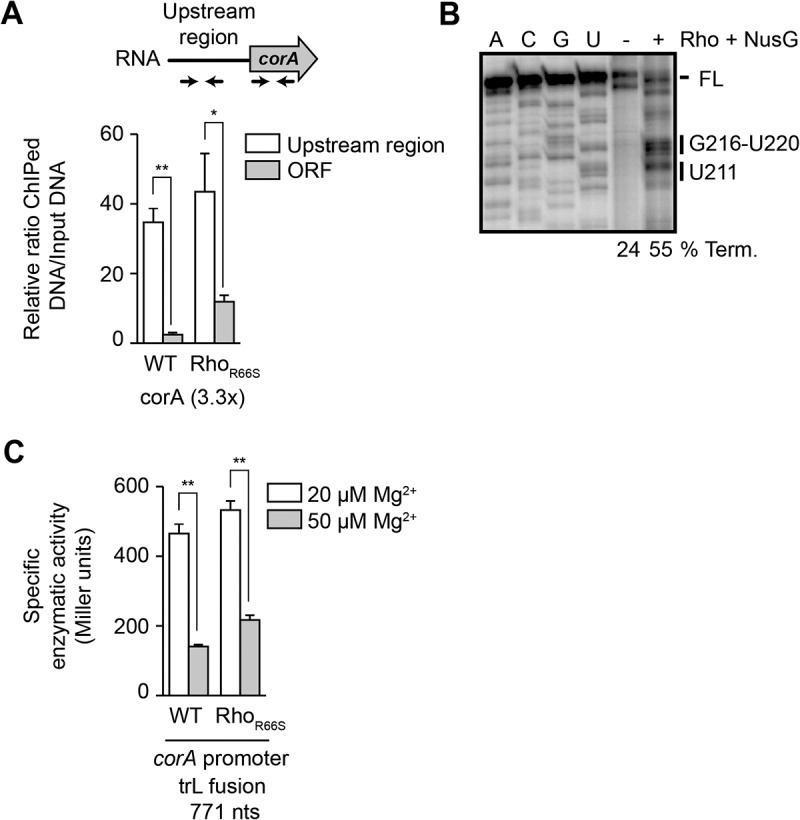
Rho-dependent transcription termination is involved in *corA* expression.

It was previously reported that Rho terminates transcription within the *corA* 5' UR in *S. enterica* [15,16]. To determine if Rho regulates *corA* expression in *E. coli* as well, we first performed chromatin immunoprecipitation (ChIP) assays of the RNAP coupled with a quantitative PCR. We previously used this technique to monitor the role of Rho in the regulation of *E. coli* riboswitches [23]. We performed these assays on the wild-type (WT) and the Rho R66S mutant strain (R66S), the latter carrying a Rho mutant defective in its ability to bind RNA [24]. To detect the amount of RNAP, we used two specific pairs of oligonucleotides: one pair was used to detect RNAP in the 5' UR and the other was used to detect RNAP in the ORF (Figure 3(A)). In the WT strain, we detected a high density of RNAP in the 5'UR and a low density in the ORF, suggesting that the 5” UR is very efficiently transcribed compared to the ORF. Importantly, in the Rho R66S mutant strain, we observed a higher proportion of RNAP in the ORF compared to the WT (Figure 3(A)), thus suggesting that Rho inhibition allows more transcription elongation through the ORF. To quantify the effect of the R66S Rho mutant on RNAP association across *corA*, we calculated a ‘Rho termination score’ [23] that is defined by the ratio of RNAP association in the ORF versus the 5' UR, normalized to the equivalent ratio in the WT. Using this approach, we found a Rho termination score of 3.3 for *corA* (Figure 3(A)). This ratio is similar to that of the Rho-regulated *E. coli thiM* riboswitch (ratio of 2.8) [23], suggesting that Rho modulates *corA* transcription.

(A) Chromatin immunoprecipitation (ChIP) assays monitoring the RNA polymerase (RNAP) occupancy. Values represent the relative enrichment of ChIP DNA to the input control in wild-type cells or cells expressing the R66S Rho mutant from its native locus. Above the plot is a schematic representation of the *corA* gene with the position of the DNA oligonucleotides. The Rho termination score (~3.3-fold) is calculated by the ratio of RNAP association in the ORF versus the 5' upstream regions in the Rho mutant normalized to the equivalent in the wild-type (ORF R66S/5' upstream region R66S)/(ORF WT/5' upstream region WT). (B) Rho termination transcription assays using the *corA* leader region. *In vitro* transcriptions were performed with and without 50 nM Rho +50 nM NusG. The full-length (FL) and Rho termination sites are indicated to the right of the gel. Sequencing lanes (A, C, G and U) have been used to map the termination sites. Termination efficiencies are indicated below the gel. (C) β-galactosidase assays of the P_corA_-*UR*_*WT*_-*corA*_*771*_-*lacZ* fusion in minimal media with 20 µM and 50 µM MgSO_4_. Experiments were performed in the wild-type (WT) and Rho-deficient R66S (R66S) strains. The average and the standard deviations are shown. Strains used in this experiment are FQ301 and FQ302.

To more directly investigate the Rho-dependent transcription termination mechanism, we performed *in vitro* transcription assays to map the site of termination. *In vitro* transcription reactions were performed in single-round conditions [25] using a DNA template containing the *corA* 5' UR and the first 18 nts of *corA* coding region. When transcription reactions were carried out using a saturating magnesium ion concentration, termination products were observed with Rho and the NusG transcription factor (Figure 3(B)). Sequencing reactions revealed that the shorter transcripts corresponded to positions U211 and G216-U220 (Figure 3(B)) in the 5' UR. Quantification of the intensity of the terminated transcripts revealed termination efficiencies of ~24% and ~55% in the absence and presence of Rho/NusG, respectively. Together, our data suggest that Rho promotes transcription termination within the 5' UR at positions similar to those previously reported for *S. enterica corA* mRNA [15].

At last, we monitored *corA* expression using ß-galactosidase assays both in the context of the WT and the Rho R66S strain. While *corA* expression was reduced by ~3.3-fold when increasing the magnesium ions concentration to 50 µM in the WT strain (Figure 3(C)), the regulation was reduced to ~ 2.4-fold in the R66S mutant strain. These results suggest a role for Rho in the Mg^2+^-dependent regulation of *corA*, and additional factors, such as ribonucleases, could be involved as well [26].

### Regulation of *corA* relies on the formation of two distinct secondary structures by the 5' UR region

To investigate the importance of *corA* 5' UR structures for regulation, we prepared two translational LacZ fusions in which either the ON or OFF state mutant is stabilized (Supplementary Figure S4). While the design of the OFF state mutant aimed at the stabilization of the stem A, the ON state mutant was expected to stabilize stem C.

**Figure 4.**
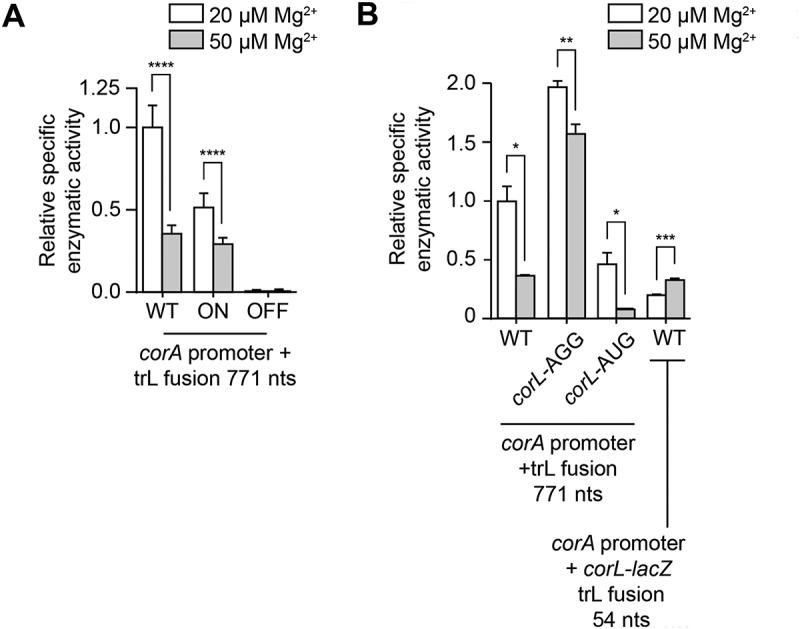
The structure of *corA* 5' upstream region and *corL* translation efficiency are important for gene regulation.

When monitoring the expression of the ON state mutant construct, we found that Mg^2+^-dependent *corA* regulation was less efficient compared to the WT (1.8-fold vs 2.8-fold, respectively) (Figure 4(A)), consistent with structural changes in the 5' UR being important for regulation. In addition, we observed that the expression of the ON state mutant at the low magnesium concentration was reduced compared to the WT (1.9-fold less), suggesting that the mRNA is less expressed in this context. Next, when monitoring the OFF state mutant, we observed that *lacZ* expression was severely decreased (Figure 4(A)), in agreement with the OFF state RNA structure inhibiting *corA* expression. Together, our results indicate that the structure of the 5' UR adopts two different secondary structures dictating the expression of *corA*.

(A) β-galactosidase assays of the P_corA_-*UR*_*WT*_-*corA*_*771*_-*lacZ*, P_corA_-*UR*_*ON*_-*corA*_*771*_-*lacZ* and P_corA_-*UR*_*OFF*_-*corA*_*771*_-*lacZ* translational fusions in a minimal media with 20 or 50 µM MgSO_4_. Values were normalized to the activity obtained in low magnesium of the wild-type fusion. The average and the standard deviations are shown. Schematics of the constructs are shown in the Supplementary Figure S4. (B) β-galactosidase assays of the P_corA_-*UR*_*WT*_-*corA*_*771*_-*lacZ*, P_corA_-*UR*_*AGG*_-*corA*_*771*_-*lacZ*, P_corA_-*UR*_*AUG*_-*corA*_*771*_-*lacZ* and P_corA_-*UR*_*54*_-*lacZ* translational fusions in a minimal media with 20 or 50 µM MgSO_4_. The average and the standard deviations are shown. Schematics of the constructs are shown in the Supplementary Figure S5. Strains used in these experiments are FM02, MG2343 and ASVB05 (panel A), and FM02, MG2298, MG2321 and MG2338 (panel B).

### Translation of *corL* uORF inhibits *corA* expression

It has been reported that uORFs play an important role in gene regulation mechanisms [10–12,27]. Regulation of *mgtA* in *S. enterica* [12] relies on the *mgtL* uORF to modulate Rho termination [11,12]. *corA* also possesses a uORF (*corL*) located in the 5' UR [15,16]. In *S. enterica*, it was reported that *corL* translation leads to Rho termination and repression of *corA* expression [15].

To establish if *corL* translation is important for the magnesium-dependent regulation of *corA*, we generated a CorA-LacZ translational construct in which the UUG start codon of *corL* was replaced with an AGG, thereby preventing translation initiation (Supplementary Figure S5). When using this construct, we found that *corA* expression was significantly increased regardless of the magnesium ion concentration (Figure 4(B)), suggesting that the translation of *corL* and *corA* is inversely proportional, as previously reported [15]. In agreement with this, replacing the *corL* start codon for the more efficient AUG codon reduced the expression of the *corA-lacZ* fusion (Figure 4(B)). For this mutant, a reduction of ~ 6-fold was observed when using 50 µM Mg^2+^ (Figure 4(B)), indicating that *corA* regulation is still efficient, while *corA* expression at 50 µM Mg^2+^ was reduced only about 1.3-fold in the *corL*-AGG mutant (Figure 4(B)). Together, our results are consistent with a previous study indicating that the efficiency of *corL* translation is important for *corA* expression [15] and magnesium regulation.

Given that *mgtL* expression is modulated by Mg^2+^ variations [12], we next investigated whether *corL* expression is also affected by changes in Mg^2+^ concentrations. To do so, we engineered a *corL-lacZ* translational fusion and monitored the ß-galactosidase activity at 20 µM and 50 µM Mg^2+^ (Figure 4(B)). When performing these experiments, we determined that the expression of *corL* was increased by ~ 1.7-fold at 50 µM Mg^2+^ (Figure 4(B)), indicating that *corL* expression is modulated by the Mg^2+^ concentration. Thus, these results indicate that an elevation of the Mg^2+^ concentration leads to a respective increase and decrease of *corL* and *corA* expression, similarly to what was reported for *mgtL* and *mgtA* expression [10–13].

### *corA* expression is modulated by intracellular changes of magnesium ions

**Figure 5.**
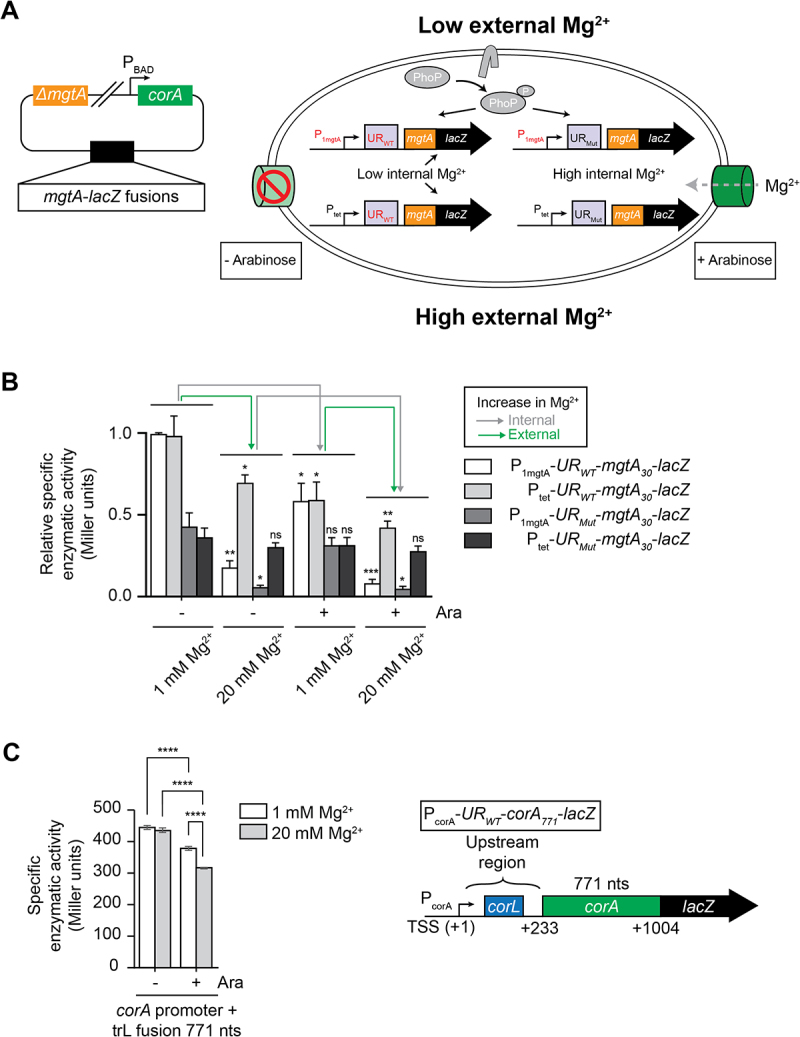
*corA e*xpression is affected by variations of internal magnesium ion concentrations.

The finding that *corA* expression is modulated in response to magnesium prompted us to investigate whether this control was mediated by external or periplasmic (Mg^2+^_e_) and/or cytoplasmic (Mg^2+^_i_) ions. For this, we took advantage of data obtained in *S. enterica* showing that free Mg^2+^_i_ remains in a limited range when Mg^2+^_e_ is changed from 0 to 20 mM, but strongly increases with Mg^2+^_e_ when CorA is overexpressed [28]. We adapted this experimental system to *E. coli* by creating a strain where the *mgtA* gene is deleted, while the *corA* promoter is replaced by an arabinose-inducible P_BAD_ promoter (Figure 5(A), left panel). Therefore, in the absence of arabinose, the strain is expected to produce only limited amounts of proteins involved in magnesium uptake, so Mg^2+^_i_ will remain relatively low. In contrast, in the presence of arabinose, CorA will be overproduced and cells will efficiently uptake magnesium, i.e. intracellular magnesium is expected to increase upon arabinose addition.

(A) *Left*, schematics of the background strain used for these assays. The strain contains a deletion of *mgtA* and relies on the P_BAD_ arabinose-dependent promoter for *corA* expression. *Right*, schematics of the four different translational fusions used to detect the magnesium concentration. The region including the 5' upstream region mutations is indicated in red and the mutant is described in the Supplementary Figure S6. (B) β-galactosidase assays using the P_1mgtA_-*UR*_*WT*_-*mgtA*_*30*_-*lacZ*, P_tet_-*UR*_*WT*_-*mgtA*_*30*_-*lacZ*, P_1mgtA_-*UR*_*Mut*_-*mgtA*_*30*_-*lacZ* and P_tet_-*UR*_*Mut*_-*mgtA*_*30*_-*lacZ* translational fusions. Experiments were performed in low (1 mM) or high (20 mM) MgSO_4_ with or without 0.2% arabinose. Values were normalized to the activity obtained in LB +1 mM MgSO_4_ using the P_1mgtA_-*UR*_*WT*_-*mgtA*_*30*_-*lacZ* fusion. The average and the standard deviations are shown. Statistical significances were calculated for each strain in each condition by comparing with the activity of the same strain obtained at 1 mM Mg^2+^. Strains used in this experiment are MG1679, MG1678, MG1708 and MG1711. (C) β-galactosidase assays using the P_corA_-*UR*_*WT*_-*corA*_*771*_-*lacZ* translational fusion (strain FM08) performed in low (1 mM) or high (20 mM) MgSO_4_ with or without 0.2% arabinose. The average and the standard deviations are shown. The construct is shown on the right of the histograms.

As a proof of concept, we first combined these *mgtA* deletions and P_BAD_-*corA* alleles with a set of four different *mgtA-lacZ* fusions responding to periplasmic and/or internal magnesium variations, based on [14] (Figure 5(A), right panel). These four fusions are at the *lacZ* locus and encode a fusion protein between the first 10 MgtA codons and ß-galactosidase; they are expressed either from the P1 promoter of *mgtA* that is under the control of PhoQ-PhoP TCS, or from the constitutively expressed P_tet_ promoter. Furthermore, they carry either the WT 5' UR region of *mgtA*, or a mutant that no longer responds to cytoplasmic Mg^2+^ ([14], see Supplementary Figure S6A and S6B for details). As expected, expression of the *mgtA-lacZ* fusion transcribed from its own promoter and carrying the WT 5' UR region (P_mgtA_-UR_WT_) displayed the largest change in expression in response to Mg^2+^ and arabinose: it was decreased ~ 5.4-fold in the presence of 20 mM Mg^2+^, ~1.7-fold by addition of 0.2% arabinose, and ~ 11-fold in 20 mM Mg^2+^ and arabinose (Figure 5(B)). In contrast, the activity of the *mgtA-lacZ* fusion expressed from the P_tet_ promoter and carrying a mutant form of the 5' UR (P_tet_-UR_Mut_) was not significantly changed in all four conditions (~1.3-fold at most). Furthermore, the fusion carrying the PhoP-dependent *mgtA* promoter, but the mutant version of the 5' UR (P_mgtA_-UR_Mut_) responded only to the addition of Mg^2+^ (~6.7-fold and ~ 5.8-fold decrease in expression in 20mM Mg^2+^ compared to 1mM Mg^2+^, without and with arabinose, respectively). No significant response was observed for this fusion when only adding arabinose , consistent with control by periplasmic Mg^2+^ only. Conversely, the fusion carrying the P_tet_ promoter and the WT 5' UR sequence (P_tet_-UR_WT_) responded to arabinose (i.e. to a change in internal magnesium concentrations) as efficiently as the P_mgtA_-UR_WT_ fusion (~1.7-fold for both construct), but much less efficiently to an increase in Mg^2+^ in the growth medium (~1.4-fold vs ~ 5.4-fold for P_tet_-UR_WT_ and P_mgtA_-UR_WT_, respectively). Together, these data validate the functionality of this assay in modulating periplasmic and internal magnesium concentrations.

Next, to assess whether *corA* expression was sensitive to periplasmic and/or internal Mg^2+^, we followed the activity of the *corA-lacZ* fusion in this ∆*mgtA* and P_BAD_-*corA* background in LB supplemented with 1 mM or 20 mM Mg^2+^, with and without addition of arabinose. In this case, arabinose led a stronger decrease in *corA* expression (~1.4-fold repression) than addition of Mg^2+^ (Figure 5(C)). This indicates that this construct responds to changes in internal Mg^2+^ concentration. When using a reporter expressed from a P_tet_ promoter (Supplementary Figure S6C), we found that the expression of the construct was also decreased when adding arabinose, more efficiently at 20 mM Mg^2+^, suggesting that this construct is also sensitive to internal Mg^2+^, even though it is slightly less responsive. Together, these results show that the expression of *corA* is modulated through the 5' UR by sensing changes in intracellular Mg^2+^ concentrations.

## Discussion

During our study, we have obtained experimental evidence that the *corA* 5' UR is important for the regulation of *corA* and is sensitive to variations in intracellular magnesium ions. Several observations are available from the literature suggesting that *corA* regulation exhibits key aspects that are like those found in the Mg^2+^-dependent control of *mgtA* expression. First, like *mgtA*, *corA* belongs to a small group of genes encoding transport systems that facilitate the influx of Mg^2+^ ions [19,29]. Second, the overall 5' UR secondary structure of *corA* is very similar to the one of *mgtA* in which a conserved three-way RNA junction is located in the 5' part of the transcript and is followed by additional stem-loop structures (Figure 1(B)) [7,15]. Furthermore, as observed for *mgtA*, the *corA* 5' UR may fold into a competing structure (Figure 1(B)), consistent with the RNA being able to adopt two mutually exclusive structures. Third, both *mgtA* and *corA* encode within the three-way RNA junction a uORF that is crucial for gene regulation [10–12,15]. Together, these observations suggest intracellular changes in Mg^2+^ ions modulate the expression of *corA*. Given that the Mg^2+^ sensing mechanism is still uncharacterized, it remains to be understood whether additional factors are involved in the regulation and to which extent it is similar to that of *mgtA*.

Since several key regulatory and structural aspects are shared between *mgtA* and *corA*, it suggests that the expression of both genes may be modulated by similar mechanisms. Here, using reporter gene assays, we provide experimental data showing that similarly to *mgtA*, expression of *corA* is sensitive to small intracellular changes of magnesium ions. Importantly, we found that while expression of *corA* is not regulated by magnesium ions during exponential growth phase, clear regulation is observed at the beginning of stationary phase (Figure 2(C)). This suggests that cellular conditions specific to the stationary phase are required for *corA* regulation. In agreement with this, it was previously determined that the magnesium/protein ratio decreases by ~2-fold in stationary phase when *E. coli* cells are grown in magnesium-limited conditions [30]. This supports the hypothesis that the lower concentration of magnesium ions in the stationary phase allows *corA* to sense subtle changes in magnesium ions concentrations. In addition, our data indicate that the expression of *corA* is sensitive to changes in Mn^2+^ concentrations (Figure 2(F)), which is in contrast to *mgtA* [7]. Thus, our data do not support previous reports suggesting that *corA* expression is constitutive [18,19] but rather indicate that the expression is modulated by changes in ionic concentrations.

Studies of *mgtA* and *corA* regulation in *S. enterica* have highlighted a key role for their respective uORF, *mgtL* and *corL* [10–12,15]. Because *corA* and *mgtA* are repressed when their respective uORF is efficiently translated [7,10,15], it suggests that efficient uORF translation is achieved at high Mg^2+^ concentrations in order to downregulate expression of *mgtA*/*corA*. It is intriguing that the RNA structure associated with high Mg^2+^ ion concentration – and efficient uORF translation – is predicted to contain the stem A that sequesters the uORF SD sequence (Figures 1(A,B)). Thus, for the translation of the uORF to be achieved, it is expected that ribosome association occurs cotranscriptionally before transcription of the stem A 3' strand is completed. Furthermore, a regulatory mechanism was proposed in which uORF translation allows access to a *rut* site, which would result in the premature Rho-dependent transcription termination of *mgtA* and *corA* in *S. enterica* [8,16]. However, it was shown for *mgtA* that only the Mg^2+^-dependent RNA folding is sufficient to modulate Rho-dependent transcription termination [8], suggesting that multiple mechanisms may be involved to maximize the sensing of Mg^2+^ ions.

No evidence of Rho-dependent transcription termination has been detected in *E. coli* for *mgtA* regulation [23], indicating that different mechanisms may be used to regulate *mgtA* in *S. enterica* and *E. coli*. In agreement with previous results [15,31], the ChIP-qPCR data revealed a Rho termination score of 3.3 for *corA* regulation (Figure 3(A)), providing additional experimental evidence for the role of Rho in *corA* regulation. Given that the ChIP-qPCR experiments were done in LB media at mid-exponential growth phase, it suggests that magnesium ions are not limiting and that Rho transcription termination is most efficient in this condition. In agreement with our data (Figure 3(B)), Rho-dependent transcription termination has been shown to occur at position ~ 220 within the leader of *S. enterica corA* and also in the vicinity of the start codon [15]. Similar Rho termination sites were found for *S. enterica mgtA* [8], suggesting that both 5' UR employ comparable strategies to regulate Rho transcription termination.

Recent experiments in *E. coli* or *Salmonella* showed that the presence of multiple prolines and acidic amino acids in *mgtL* destabilizes its translation and that higher concentrations of Mg^2+^ ions may counteract the destabilizing effect [12,13]. The r-protein bL31 is required for this control of *mgtL* translation by Mg^2+^ and, consistent with *mgtL* translation leading to repression of *mgtA* transcription, deletion of bL31 increased MgtA levels by 13-fold [13]. CorA was more expressed as well (2-fold) in the delta bL31 strain, suggesting that *corL* translation could also be impaired in the absence of bL31. Although it is possible that such an intrinsic destabilization mechanism of the ribosome occurs when translating *corL*, it appears that there are fewer amino acids either being a proline or acidic, consistent with a lower destabilization effect (~2-fold) during *corL* translation [13]. In this study, our results are in very good agreement with the expression of *corL* and *corA* being inversely related. For instance, *corA* expression is reduced both with the introduction of a better start codon (AUG) and in the ON state mutant that favors the access to the *corL* SD sequence (Figures 4(A,B)). However, the use of a non-initiating codon (AGG) resulted *corA* to be significantly overexpressed (Figure 4(B)). It is most probable that the presence of a non-start codon results in the stalling of the small ribosomal subunit at the beginning of *corL*, thus allowing to modulate the RNA structure in the ON state. Furthermore, the mechanism underlying the remaining response to Mg^2+^ in the *corL*-AGG background (1.3-fold) is unknown. This could be due to a residual, weak, translation from an alternative start codon in the *corL* sequence (for instance, the internal CUG codon) or, alternatively, this modest regulation could be independent from *corL* translation. These results suggest that *corA* expression is increased by inefficient elongation of *corL* translation. Furthermore, despite the bulk of our data suggesting that *corA* expression is regulated by changes in Mg^2+^ concentrations, some important aspects are still not fully understood. For example, magnesium ions could have a similar role as found in *mgtA* regulation where the efficiency of *mgtL* translation elongation is affected [13]. In such a scenario, the presence of acidic and proline codons in *mgtL* makes its translation intrinsically inefficient [10,12,13], thus allowing the leader to adopt the ON state and efficient *mgtA* expression (Figure 1(A)). However, at higher Mg^2+^ concentrations, *mgtL* translation elongation is more efficient resulting in the leader folding into the OFF state (Figure 1(A)) and thus the inhibition of *mgtA* expression. Interestingly, it was reported that the N-terminal region of *corL* is conserved and contains a single proline residue [15], suggesting that the translation of *corL* may not rely as strongly on the presence of proline residues as *mgtL* for the regulation of *corA*. In addition, it is uncertain whether magnesium ions play a role in changing the structure of the *corA* leader and, if they do, at which point in the regulatory process. For example, magnesium ions could be involved at the very first step of the regulation by selectively controlling the initiation of *corL* translation by modulating the leader structure.

**Figure 6.**
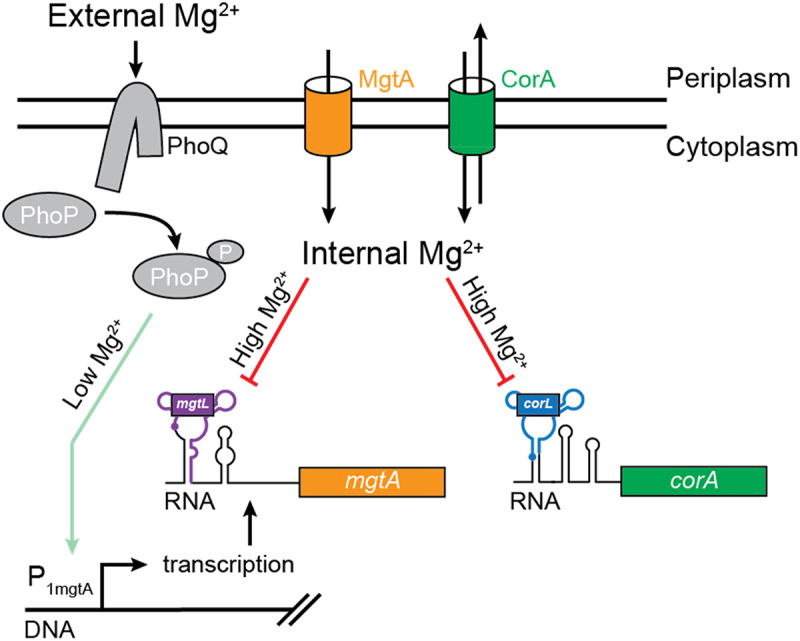
External and internal magnesium ions differently modulate the expression of *mgtA* and *corA.*

Our study shows that in *E. coli*, the expression of *mgtA* and *corA* is affected by different sources of magnesium ions. Using a mutant strain in which the entry of magnesium ions is controlled by the addition of arabinose (inducing the production of the CorA transporter), we were able to determine that the regulation of *mgtA* and *corA* is affected differently by extracellular and intracellular magnesium ions (Figure 5). This is mostly due to the PhoQ/PhoP system controlling the promoter activity of *mgtA* but not of *corA*. In such a model (Figure 6), by virtue of the PhoQ sensor, a low extracellular concentration of magnesium ions leads to the activation of the *mgtA* promoter via the phosphorylation of PhoP. The production of the MgtA transporter leads to a greater intracellular entry of magnesium ions via the MgtA transporter. Providing that a saturating Mg^2+^ concentration is reached, the *mgtA* 5' UR adopts the OFF state conformation and downregulates *mgtA* expression via Rho-dependent transcription termination. In contrast, since the *corA* promoter is constitutively active and does not rely on the PhoQ/PhoP system, *corA* expression is only mediated by the 5' UR and is therefore only modulated by changes in the intracellular concentration of magnesium ions (Figure 6). In this model, it is expected that *corA* regulation relies on a mechanism similar to that observed for *mgtA* [12], which would imply that elevated Mg^2+^ concentrations could increase the efficiency of *corL* translation, and therefore result in the repression of *corA* via Rho-dependent transcription termination. Consequently, the *corA* regulation mechanism is unique because it is not regulated at the promoter level like *mgtA* but instead is solely controlled by the leader ability to sense intracellular magnesium ions [12,15].

At low extracellular concentrations of magnesium ions, the PhoQ sensor phosphorylates PhoP, which leads to the activation of the *mgtA* promoter (green arrow), production of the *mgtA* transcript and translation of the MgtA transporter. However, when the intracellular concentration of magnesium ions has reached sufficiently high levels (red lines), the leader regions of *mgtA* and *corA* transcripts turn off the expression of downstream genes. Consequently, while the expression of *mgtA* is regulated by the extra- and intracellular concentrations of magnesium ions, the expression of *corA* is only affected by variations of intracellular magnesium ions. The promoter region of *corA* is not shown since it is not affected by magnesium ions.

In conclusion, our data show that *corA* expression is influenced by magnesium ions during the stationary phase of bacterial growth and that both intracellular and extracellular magnesium ions affect *corA* and *mgtA* expression in different ways. Specifically, the expression of *corA* only responds to changes in intracellular magnesium ions, which allows the bacteria to maintain cellular homeostasis.

## Materials and methods

### Strains and general microbiology techniques

Strains used in this study are derived from *E. coli* MG1655. Strain genotypes are listed in Supplementary Table S1. The different *lacZ* fusions and mutant alleles were constructed by recombineering PCR products. The preparation of the different constructs is described in Supplementary Table S2. DNA oligonucleotides used in this study are listed in Supplementary Table S3. To follow the response of *corA* to magnesium ions, the strains were grown in M63 minimal medium with 0.2% glucose and different MgSO_4_ concentrations, unless otherwise indicated. The double mutants *∆mgtA* and P_BAD_-*corA* . were maintained in LB medium supplemented with at least 20 mM MgSO_4_

### Strain constructions

The *corA* and *mgtA* deletion mutants were constructed using lambda Red recombineering in the strain NM300 with a mini-lambda (received from N. Majdalani, NIH) as previously described [32]. The PCR products used a gentamycin- or erythromycin-resistance gene flanked by homology regions to *corA* or *mgtA* external sequences, respectively. They were amplified from a plasmid DNA template and were treated with DpnI prior to recombineering. After selection (using erythromycin at 200 µg/ml or gentamycin at 10 µg/mL), purification, and validation by sequencing, the mutant alleles were then moved by P1 transduction as needed. Similarly, the GenR-P_BAD_-*corA* allele was made by recombineering to insert a GenR-P_BAD_ construct upstream of the *corA* transcription start site, validated by sequencing and transduced in other strains as necessary.

Fusions with *lacZ* were made by recombineering PCR products into the strain MG1508 that carries a Ptet-*cat-sacB-lacZ* construct allowing counter-selection on sucrose and Xgal plates. Fusions were systematically checked by sequencing. The mutation in the leader region of *mgtA-lacZ* fusions (strains MG1532, MG1534 and derivatives) consists in a change from GUAAGGCCGUGA to AAUUAUAAUAAU of nts 152–163 of *mgtA* 5' UR and a deletion of residue C175 (relative to the + 1 TSS).

### β-galactosidase assays

Kinetic assays for β-galactosidase experiments were performed as described previously [33]. Briefly, an overnight bacterial culture grown in M63 0.2% glucose minimal medium was washed two times with 1X salt solution and diluted to an OD_600_ of 0.02 in 50 ml of fresh medium with 20 or 50 µM MgSO_4_. The culture was incubated at 37°C and samples were taken at 3 h and 5.5 h up to 9 h, each 30 min. For the intra/extracellular magnesium variation, an overnight bacterial culture grown in LB, supplemented with 1 mM or 20 mM MgSO_4_ was diluted to an OD600 of 0.02 in 50 mL of fresh medium with 1 mM or 20 mM and with/without arabinose (0.2%). Samples were taken at OD_600_ 0.4. The experiments were performed in triplicate and the average values with standard deviations are reported.

For Miller assays, overnight cultures in M63 medium with 0.2% glucose and 100 µM MgSO_4_ were diluted 200-fold in fresh medium with the indicated MgSO_4_ concentration. After 7 h of growth, 200 µL of cells were mixed with Z buffer and the ß-galactosidase activity was assayed as previously described [34]. In the case of the *∆mgtA* and P_BAD_-*corA* double mutants that display a strong dependency to Mg^2+^ (Figure 5), cells were grown overnight in LB supplemented with 1 mM MgSO_4_, and diluted 200-fold in the indicated media. Cells were collected for ß-galactosidase assays when the absorbance of the culture at 600 nm reached 0.4.

All experiments were done in triplicate and the average ß-galactosidase activities and standard deviations are shown. The statistical significance of the results was systematically assessed with a bilateral heteroscedastic Student t-test (****: P-value <5 × 10^−5^; ***: P-value <5 × 10^−4^; **: P-value <0.005; *: P-value <0.05).

### RNA extraction and northern blot analysis

Overnight cultures of WT and *∆corA* strains in M63 minimal medium with 0.2% glucose and 100 µM MgSO_4_ were diluted 200-fold in the same medium but containing 20 µM or 50 µM MgSO_4_.When the absorbance at 600 nm reached 1.5, cells were pelleted by centrifugation and resuspended in 1X PBS. After three extractions with phenol/chloroform/isoamyl alcohol, total RNA was precipitated and resuspended in water. An amount of 5 µg of total RNA was then resolved on a 1% agarose gel in 1X TBE, transferred to a nitrocellulose membrane (Hybond N+, Amersham) and *corA* mRNA was detected using a specific biotinylated probe as previously described [35].

### ChIP-qPCR

Wild-type MG1655 and MG1655 *rho*R66S mutant strains were grown in Luria broth (LB) at 37°C to mid-exponential phase. ChIP of RNAP was performed as described previously [23] using anti-ß mouse monoclonal antibody (Neo-Clone). After purification of ChIP and input (starting material) DNA samples, real-time PCR was performed using an ABI 7500 instrument with the primers listed in Supplementary Table S3. In each reaction, forward and reverse primers were used to amplify either the UTR or ORF region of *rho*. The percentage of immunoprecipitation (IP) efficiency was calculated for each region of interest as the ratio of DNA in the ChIP sample compared to the input sample. The statistical significance of the results was systematically assessed with a bilateral heteroscedastic Student t-test (****: P-value <5 × 10^−5^; ***: P-value <5 × 10^−4^; **: P-value <0.005; *: P-value <0.05).

### Rho-dependent transcription termination assays

*In vitro* transcriptions were performed using the six codons construct. The transcription termination assays were performed as described previously [23]. In a reacting vessel, 2 pmol DNA template, 10 µM GAU trinucleotide and 10 µM ATP/CTP and [α-^32^P] UTP were incubated in the transcription buffer at 37°C for 5 minutes. Rho (100 nM) and NusG were added in the first step of the transcription reaction. The transcription reaction was performed as described [23] with a solution containing 1 mM NTPs and 20 µg/mL rifampicin. The resulting reactions were phenol/chloroform treated and mixed 2:1 with a stop solution. Rho and NusG proteins were purified as described previously [36]. Transcriptional sequencing was performed by including 3'-O-methyl NTP in the reactions at varying concentrations to only permit a small fraction of transcriptional arrests [37].

## Supplementary Material

Supplemental Material

## Data Availability

The data supporting the findings of this study are available within the article and its supplementary materials. Strains and plasmids used in this study are available upon request to the authors.
