# Supplementary material for: Regulation of magnesium ion transport in Escherichia coli: insights into the role of the 5’ upstream region in corA expression
Source: RNA Biol. 2024 Nov 8;21(1):1171–83. doi: 10.1080/15476286.2024.2421665 (PMC11552253; doi:10.1080/15476286.2024.2421665)
Supplement: Supplemental Material [file KRNB_A_2421665_SM1221.pdf]

Regulation of Magnesium Ion Transport in *Escherichia coli*: Insights into the Role of the 5' Upstream Region in *corA* Expression

A.-S. Vézina Bédard[1], A. Michaud[1], F. Quenette[2], N. Singh[3], F. de Lemos Martins[2], J.T. Wade[3,4], M. Guillier[2*] and D.A. Lafontaine[1*]

[1]Department of Biology, Faculty of Science, RNA Group, Université de Sherbrooke, Sherbrooke, Quebec, Canada, J1K 2R1.

[2]Expression Génétique Microbienne, UMR8261 CNRS, Université Paris Cité, Institut de Biologie Physico-Chimique, 75005 Paris, France.

[3]Wadsworth Center, New York State Department of Health, Albany, NY 12208, USA

[4]Department of Biomedical Sciences, University at Albany, Albany, NY 12201, USA.

*Corresponding author. E-mail: maude.guillier@ibpc.fr, daniel.lafontaine@usherbrooke.ca

Keywords: Genetic regulation, leader region, magnesium homeostasis, regulatory RNA, *corA*

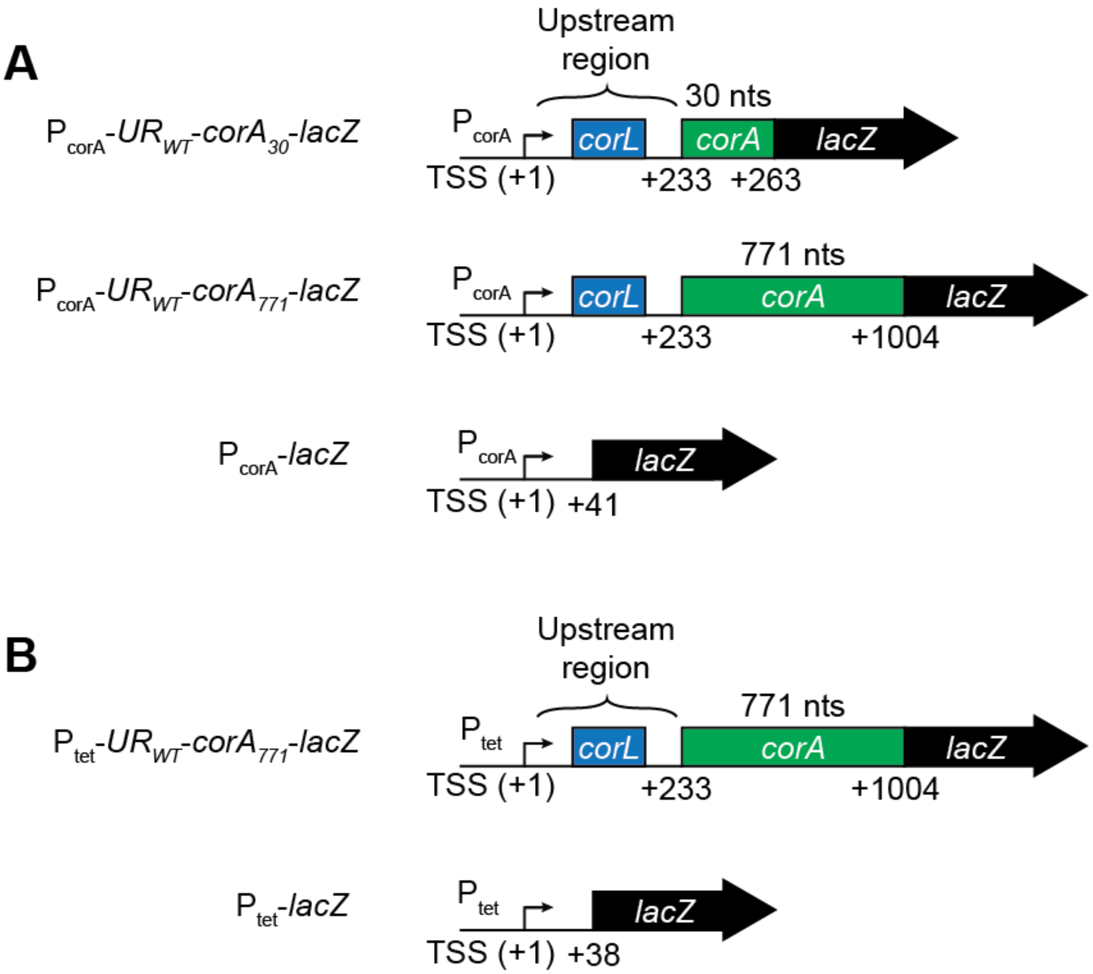

Supplementary Figure S1. Schematics of *corA-lacZ* translational fusions used to monitor *corA* expression. (A) Schematics of the P_{corA}-*UR_{WT}-$corA_{30}$-lacZ*, P_{corA}-*UR_{WT}-$corA_{771}$-lacZ* and P_{corA}-*lacZ* constructs. The transcription start site (TSS) and 5' UR region are shown. For each construct, the *corA* coding region is fused to the 28th nucleotide of *lacZ*. The 5' upstream region (UR) of the P_{corA}-*lacZ* construct is constituted by the first 24th nt of the *corA* transcript that are fused to the 17 nt upstream of the *lacZ* coding region, thus making a total of 41 nt. (B) Schematics of the P_{tet}-*UR_{WT}-$corA_{771}$-lacZ* and P_{tet}-*lacZ* constructs. The *tet* promoter sequence corresponds to the $P_{LtetO-1}$ construct (1). The transcription start site (TSS) and 5' UR are shown. For each construct, the *corA* coding region is fused to the 28th nucleotide of *lacZ*.

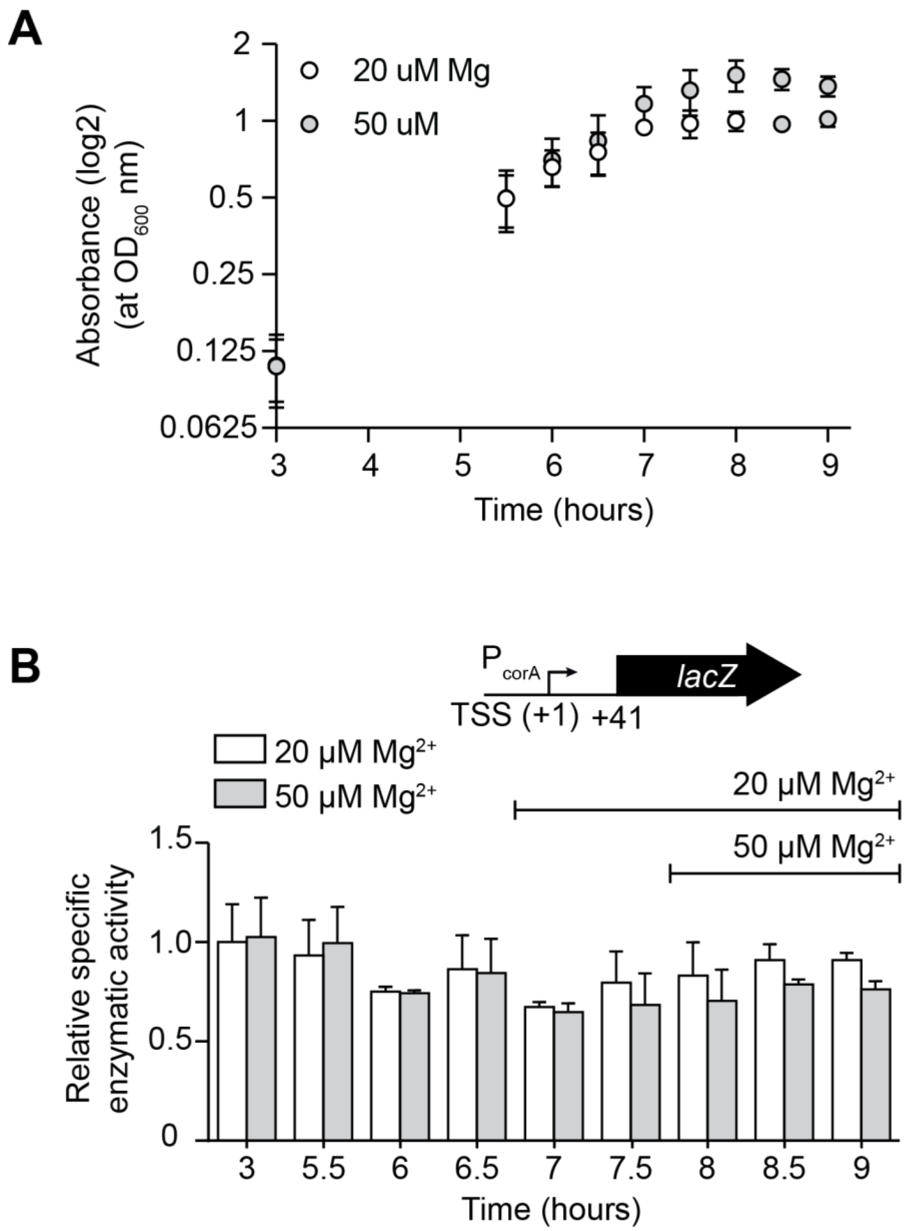

Supplementary Figure S2. The expression of *corA* is modulated by magnesium ions. (A) Cellular density monitored at 600 nm over the course of bacterial growth performed in minimal media with 20 or 50 µM $MgSO_4$. The average and the standard deviations are shown. While the growth stationary phase is attained at 7 h with 20 µM $MgSO_4$, it is reached at 8 h with 50 µM $MgSO_4$. (B) β-galactosidase assays of the P_{corA}-*lacZ* fusion in minimal media with 20 µM $MgSO_4$ and 50 µM $MgSO_4$. The horizontal bars indicate the time points corresponding to the stationary growth phase. The average and the standard deviations are shown. The construct is shown above the histograms.

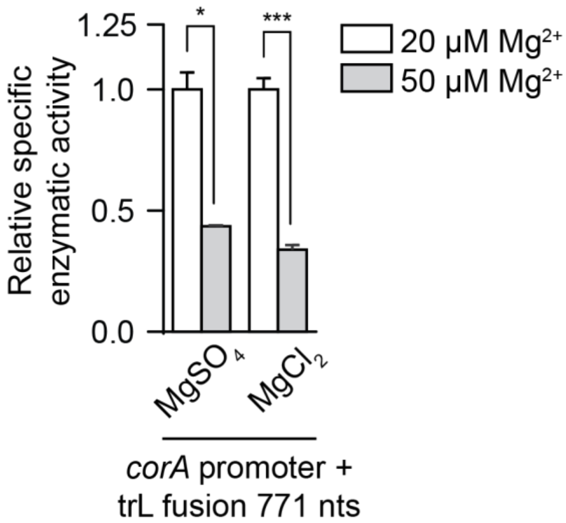

Supplementary Figure S3. The type of magnesium salt does not affect the regulation of *corA* expression. β-galactosidase assays of the P_{corA}-UR_{WT}-$corA_{771}$-*lacZ* fusions in minimal media with 20 or 50 µM $MgSO_4$ or $MgCl_2$. The average and the standard deviations are shown.

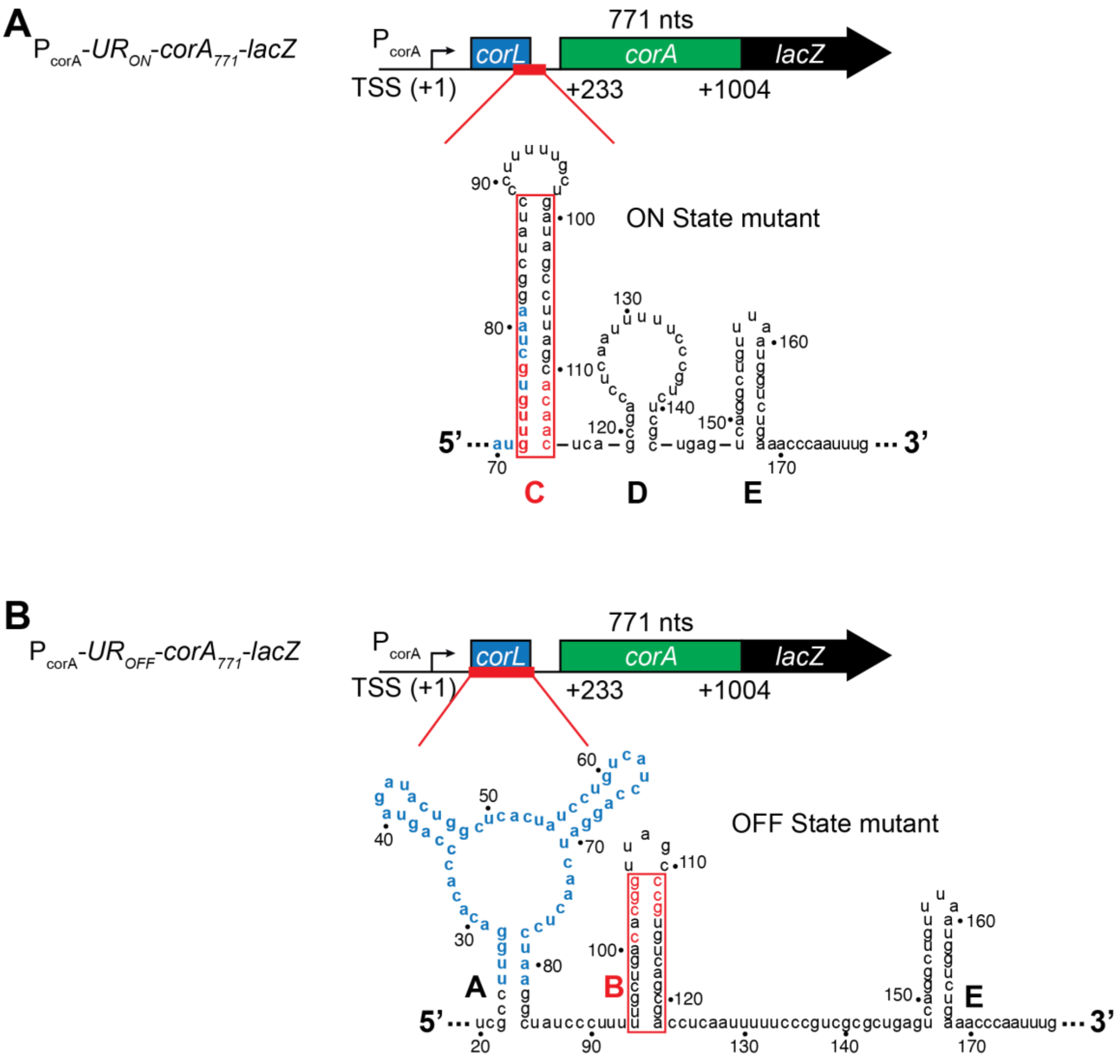

Supplementary Figure S4. Schematics of *corA-lacZ* translational fusions used to study the importance of the 5' upstream region structure on *corA* expression. (A,B) Secondary structures of the *corA* leader in the ON state (A) and OFF state (B). Mutations are indicated in red and have been inserted to favor each structural state. The translational fusions containing the mutants are shown above each structure.

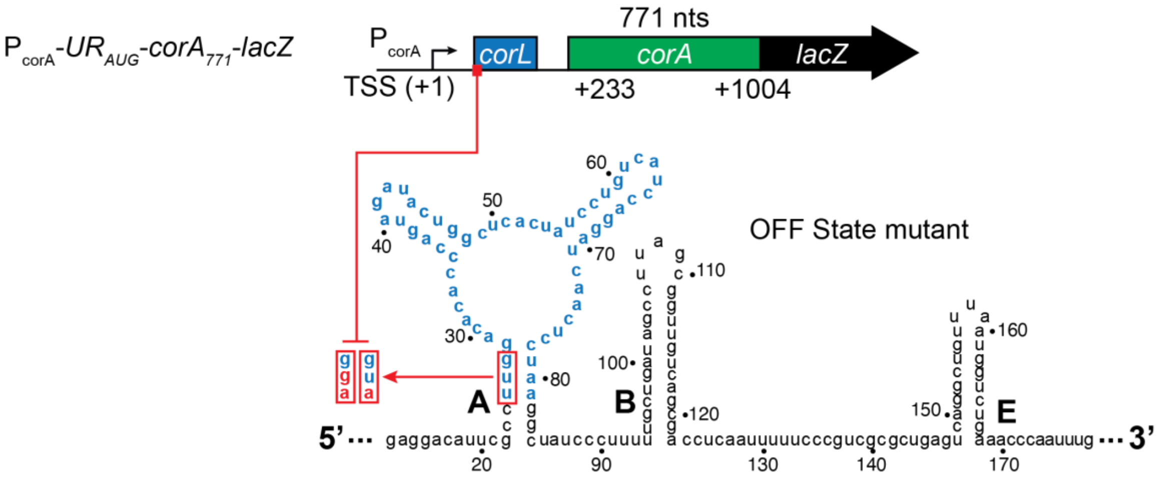

Supplementary Figure S5. Schematics of *corA-lacZ* translational fusions used to study the importance of *corL* translational activity. Secondary structures of the *corA* 5' upstream leader when the *corL* start codon is changed for AGG or AUG. Mutations are indicated in red and the translational fusion containing the mutants is shown above the structure.

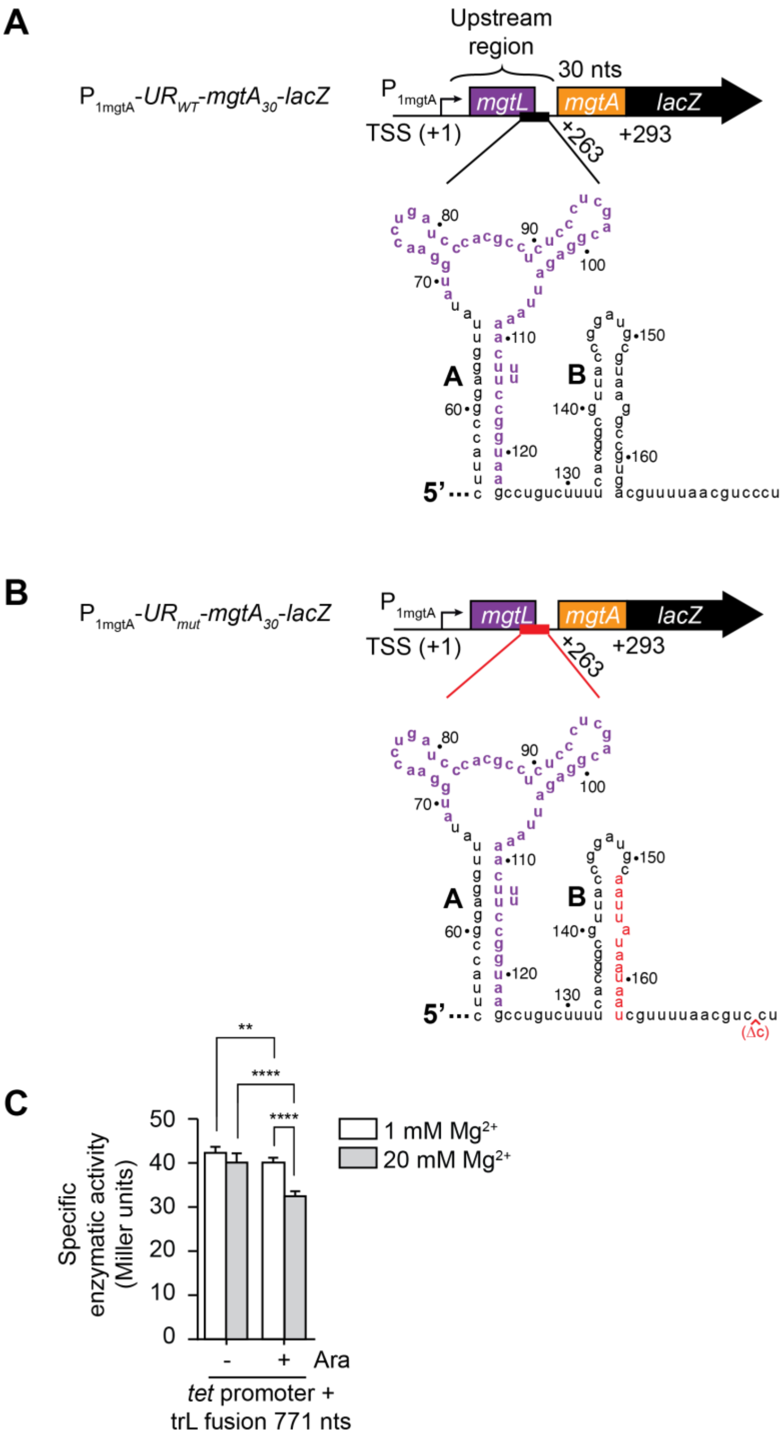

Supplementary Figure S6. Schematics of *mgtA-lacZ* translational fusions used to monitor the levels of magnesium ions in a mutant strain. (A-B) Secondary structures of *mgtA* 5' upstream region in a wild-type construct (A) or when the sequence involved in the B stem is altered (B). The mutations are indicated in red; note that a 1 nt-deletion (ΔC in red) was introduced as well during the construction of the stem B mutant. The transcription start site (TSS) is shown. (C) β-galactosidase assays using the P_{tet}-$\mathit{UR_{WT}}$-$\mathit{corA_{771}}$-*lacZ*

translational fusion performed in low (1 mM) or high (20 mM) $MgSO_4$ with or without 0.2% arabinose. The average and the standard deviations are shown.

Supplementary Table S1. Summary of strains used in this study.

Strains	Constructs	References
MG1655	WT strain	F. Blattner's Lab
DJ624	MG1655 *ΔlacX74 mal ::lacI*^{q}	D. Jin (NIH)
NM300	MG1655 *ΔlacX74 mini-lambda ::tet*^{R}	N. Majdalani (NIH)
MG1508	MG1655 *mal ::lacI*^{q} P_{tet}-*cat-sacB-lacZ, mini-lambda ::tet*^{R}	(2)
MG1525	MG1655 *mal ::lacI*^{q} P_{tet}-UR_{WT}-$mgtA_{30}$-*lacZ*	This study; Recombineering into MG1508
MG1528	MG1655 *mal ::lacI*^{q} P_{1mgtA}-UR_{WT}-$mgtA_{30}$-*lacZ*	This study; Recombineering into MG1508
MG1532	MG1655 *mal ::lacI*^{q} P_{tet}-UR_{mut}-$mgtA_{30}$-*lacZ*	This study; Recombineering into MG1508
MG1534	MG1655 *mal ::lacI*^{q} P_{1mgtA}-UR_{mut}-$mgtA_{30}$-*lacZ*	This study; Recombineering into MG1508
MG1578	NM300 *ΔcorA::gen*	This study; Recombineering *ΔcorA::gen* in NM300
MG1595	NM300 *ΔmgtA:: erm*	This study; Recombineering *ΔmgtA::erm* into NM300
MG1597	DJ624 *ΔcorA::gen*	This study; DJ624 + P1 (MG1578)
MG1634	NM300 GenR-P_{BAD}-*corA*	This study; Recombineering into NM300
MG1659	MG1655 *mal ::lacI*^{q} P_{tet}-UR_{WT}-$mgtA_{30}$-*lacZ GenR*-P_{BAD}-*corA*	This study; MG1525 + P1(MG1634)
MG1660	MG1655 *mal ::lacI*^{q} P_{1mgtA}-UR_{WT}-$mgtA_{30}$-*lacZ GenR*-P_{BAD}-*corA*	This study; MG1528 + P1(MG1634)
MG1678	MG1655 *mal ::lacI*^{q} P_{tet}-UR_{WT}-$mgtA_{30}$-*lacZ GenR*-P_{BAD}-*corA ΔmgtA ::erm*	This study; MG1659 + P1 (MG1595)
MG1679	MG1655 *mal ::lacI*^{q} P_{1mgtA}-UR_{WT}-$mgtA_{30}$-*lacZ GenR*-P_{BAD}-*corA ΔmgtA ::erm*	This study; MG1660 + P1 (MG1595)
MG1703	MG1655 *mal ::lacI*^{q} P_{1mgtA}-UR_{mut}-$mgtA_{30}$-*lacZ GenR*-P_{BAD}-*corA*	This study; MG1534 + P1(MG1634),
MG1708	MG1655 *mal ::lacI*^{q} P_{1mgtA}-UR_{mut}-$mgtA_{30}$-*lacZ GenR*-P_{BAD}-*corA ΔmgtA ::erm*	This study; MG1703 + P1 (MG1595)
MG1710	MG1655 *mal ::lacI*^{q} P_{tet}-cat-sacB-$lacZ_{+28}$ *GenR*-P_{BAD}-*corA ΔmgtA ::erm*	This study; MG1679 + P1(MG1508), selection on LB-Cmp-20mM $MgSO_4$
MG1711	MG1655 *mal ::lacI*^{q} P_{tet}-UR_{mut}-$mgtA_{30}$-*lacZ GenR*-P_{BAD}-*corA ΔmgtA ::erm*	This study; MG1710 + P1 (MG1532), selection on minimal A medium with 0,2% lactose and 50mM $MgSO_4$
MG2297	MG1655 *mal ::lacI*^{q} P_{corA}-*lacZ*	This study, recombineering into MG1508
MG2298	MG1655 *mal ::lacI*^{q} P_{corA}-UR_{AGG}-$corA_{771}$-*lacZ*	This study, recombineering into MG1508
MG2314	MG1655 *mal ::lacI*^{q} P_{corA}-UR_{WT}-$corA_{30}$-*lacZ*	This study, recombineering into MG1508
MG2316	MG1655 *mal ::lacI*^{q} P_{tet}-*lacZ*	This study, recombineering into MG1508
MG2321	MG1655 *mal ::lacI*^{q} P_{corA}-UR_{AUG}-$corA_{771}$-*lacZ*	This study, recombineering into MG1508
MG2338	MG1655 *mal ::lacI*^{q} P_{corA}-*corL-lacZ*	This study, recombineering into MG1508
MG2343	MG1655 *mal ::lacI*^{q} P_{corA}-UR_{ON}-$corA_{771}$-*lacZ*	This study, recombineering into MG1508
FM02	MG1655 *mal ::lacI*^{q} P_{corA}-UR_{WT}-$corA_{771}$-*lacZ*	This study, recombineering into MG1508
FM06	MG1655 *mal ::lacI*^{q} P_{tet}-UR_{WT}-$corA_{771}$-*lacZ*	This study, recombineering into MG1508

FM08	MG1655 *mal ::lacI^q* P_{corA}-*UR_{WT}-$corA_{771}$-lacZ GenR-P_{BAD}-corA ΔmgtA ::erm*	This study; MG1710 + P1 (FM02), selection on minimal A medium with 0,2% lactose and 50mM $MgSO_4$
FM09	MG1655 *mal ::lacI^q* P_{tet}-*UR_{WT}-$corA_{771}$-lacZ GenR-P_{BAD}-corA ΔmgtA ::erm*	This study; MG1710 + P1 (FM06), selection on minimal A medium with 0,2% lactose and 50mM $MgSO_4$
ASVB05	MG1655 *mal ::lacI^q* P_{corA}-*UR_{OFF}-$corA_{771}$*-lacZ	This study, recombineering into MG1508
OK756	MG1655 Δ*lacX74 mal::lacI^q rho-R66S*	(3)
FQ301	MG1655 *mal::lacI^q* P_{corA}-*UR_{WT}-$corA_{771}$-lacZ*	This study; DJ624 + P1 (FM02)
FQ302	MG1655 *mal::lacI^q* P_{corA}-*UR_{WT}-$corA_{771}$-lacZ rho-R66S*	This study; OK756 + P1 (FM02)

Supplementary Table S2. PCR constructs used for *in vivo* and *in vitro assays*

Strains	Constructs	Oligonucleotides (template)
***corA* and *mgtA* mutations**		
MG1578	*ΔcorA ::gen*	PCR1: 5'corA::gen – 3'corA::gen (pBBR1MCS-5 plasmid, ref: Kovach and Robertson, Gene 1995, PMID 8529885)
MG1595	*ΔmgtA ::erm*	PCR1: 5'mgtA::erm – 3'mgtA::erm (plasmid pDG641, gift from P. Stragier (4)
MG1634	*GenR-P_{BAD}-corA*	PCR1: GencorAfor - GenP_{BAD}rev (genomic DNA of a GenR strain)<br>PCR 2: Gen$P_{BAD}$for - P_{BAD}corArev (genomic DNA of a strain carrying a chromosomal P_{BAD} sequence)<br>PCR 3: GencorAfor - P_{BAD}corArev (PCR 1-2)
***lacZ* fusions in MG1508**		
MG1525	P_{tet}-*UR_{WT}-$mgtA_{30}$-lacZ*	PCR1: 5'Ptet-mgtA – 3'mgtA-lacZ (genomic DNA)
MG1528	P_{1mgtA}-*UR_{WT}-$mgtA_{30}$-lacZ*	PCR1: 5'lacI-P1mgtA – 3'mgtA-lacZ (genomic DNA)
MG1532	P_{tet}-*UR_{mut}-$mgtA_{30}$-lacZ*	PCR1: 5'Ptet-mgtA – mgtA-mutribo-rev (genomic DNA)<br>PCR2: mgtA-mutribo-for – 3'mgtA-lacZ (genomic DNA)<br>PCR3: 5'Ptet-mgtA – 3'mgtA-lacZ (PCR1-2)
MG1534	P_{1mgtA}-*UR_{mut}-$mgtA_{30}$-lacZ*	PCR1: 5'lacI-P1mgtA – mgtA-mutribo-rev (genomic DNA)<br>PCR2: mgtA-mutribo-for – 3'mgtA-lacZ (genomic DNA)<br>PCR3: 5'Ptet-mgtA – 3'mgtA-lacZ (PCR1-2)
MG2297	P_{corA}-*lacZ* (corA region (-309-209, relative to AUG) upstream of *lacZ* (from -17))	PCR1: 5' corA-309 – corAlacZ-17rev (genomic DNA)<br>PCR2: 5' corA-309 – lacZ4-67rev (PCR1)
MG2298	P_{corA}-*UR_{AGG}-$corA_{771}$-lacZ*	PCR1: 5' corA-309 – corAmutORF1rev (genomic DNA)<br>PCR2: corAmutORF1for – oFM3 (genomic DNA)<br>PCR3: 5' corA-309 – oFM3 (PCR1-2)
MG2314	P_{corA}-*UR_{WT}-$corA_{30}$-lacZ*	PCR1: 5' corA-309 – corA10-lacZrev (genomic DNA)
MG2316	P_{tet}-*lacZ*	PCR1: Ptet-lacZ-38 – lacZ28-66rev (genomic DNA)
MG2321	P_{corA}-*UR_{AUG}-$corA_{771}$-lacZ*	PCR1: 5' corA-309 – corAORF1AUGrev (genomic DNA)<br>PCR2: corAORF1AUGfor – oFM3 (genomic DNA)<br>PCR3: 5' corA-309 – oFM3 (PCR1-2)
MG2338	P_{corA}-*corL-lacZ*	PCR1: 5' corA-309 – corL-lacZR (genomic DNA)
MG2343	P_{corA}-*UR_{ON}-$corA_{771}$-lacZ*	PCR1: 5' corA-309 – corA41-71txR (genomic DNA)<br>PCR2: corAON1F – oFM3 (genomic DNA)<br>PCR3: corAON1F-reamp – oFM3 (PCR2)<br>PCR4: 5' corA-309 – oFM3 (PCR1 and 3)
FM02	P_{corA}-*UR_{WT}-$corA_{771}$-lacZ*	PCR1: 5' corA-309 – oFM3 (genomic DNA)
FM06	P_{tet}-*UR_{WT}-$corA_{771}$-lacZ*	PCR1: oFM4 – oFM3 (genomic DNA)
ASVB05	P_{corA}-*UR_{OFF}-$corA_{771}$-lacZ*	PCR1: corA-309 – corA68-99txR (genomic DNA)<br>PCR2: corAOFF1F - oFM3 (genomic DNA)<br>PCR3: corA-309 - oFM3 (PCR1-2)
***In vitro* transcription assays**		
PlacUV5-$corA_{18}$		PCR1 F-pLacUV5-corA – R-corA6codons (genomic DNA)

Table S3. Summary of oligonucleotides used in this study.

Oligonucleotides	Sequences 5'-3'
5'corA::gen	CTGTGGTGTTCTGTTGCGTGTTGACGGCAAAATTTTGCTGGGACGCACACCGTGGAAACGG
3'corA::gen	CAGGTGGCTTAGCCAGACTAAGCCACCGCTCTCGTTTTTTAGCGGCGTTGTGACAATTTACC
5'mgtA::erm	GCTACGAATATTATTGGATTCTCCTTATTATTTGCGGCGCTTTCAATAATCGCATCCGATTGC
3'mgtA::erm	CTACAATCTGAATCGGGGCTATCGTGCCCAGTTTATTCTTTAGATAACTCGGCGTATGTTATTC
GencorAfor	CGTGTTGACGGCAAAATTTTGCTGGCGTAACATGCGCGCACGACGCACACCGTGGAAACGG
GenP$_{BAD}$rev	CTGAAGCGCAAAATGATCCCCGCGGCGTTGTGACAATTTACC
GenP$_{BAD}$for	GGTAAATTGTCACAACGCCGCGGGGATCATTTTGCGCTTCAG
P$_{BAD}$corArev	CTACTGGGTGTGTCCAAGGCGAATGTCCTCTTAGAGTGATCATGGAGAAACAGTAGAGAGTTG
5' corA-309	GCGAAGCGGCATGCATTTACGTTGACACCATCGAATGGCGCCGCGAAGTTCAACTATTGTTC
lacZ4-67rev	TAACGCCAGGGTTTTCCCAGTCACGACGTTGTAAAACGACGGCCAGTGAATCCGTAATCATGGT
corAlacZ-17rev	GTGAATCCGTAATCATGGTCATAGCTGTTTCCTGTGTGAGGCGAATGTCCTCTTAGAGTG
corA10-lacZrev	TAACGCCAGGGTTTTCCCAGTCACGACGTTGTAAAACGACGTTATTTTCCAGTTGAAATGCGC
oFM3	TAACGCCAGGGTTTTCCCAGTCACGACGTTGTAAAACGACTTTGATGATGCGGTTCTGCTC
oFM4	GATAGAGATTGACATCCCTATCAGTGATAGAGATACTGAGCACGATCACTCTAAGAGGACATTC
corA41-71txR	ATCCTGGATGACAGGATAGTGAGCCAGTATC
corAON1F	AGGCTATCCCTTTTTGCTGATAGCCTTAGCACAACTCAGCGACCTCAATTTTTCCCGTCGCGC
corAON1F-reamp	GATACTGGCTCACTATCCTGTCATCCAGGATGTTGTGCTAAGGCTATCCCTTTTTGCTGATAGC
corA68-99txR	CAGCAAAAAGGGATAGCCTTAGGAGTTGATCC
corAOFF1F	GGATCAACTCCTAAGGCTATCCCTTTTTGCTGACACGGTTAGCCCGTGTCAGCGACCTCAATTTTTCCCGTCG
corL-lacZR	AACGCCAGGGTTTTCCCAGTCACGACGTTGTAAAACGACGGAGTTGATCCTGGATGACAGG
corAmutORF1rev	TGGGTGTGTCCCTGGCGAATGTCCTCTTAGAGTG
corAmutORF1for	GACATTCGCCAGGGACACACCCAGTAGATACTGG
corAORF1AUGrev	TGGGTGTGTCCATGGCGAATGTCCTCTTAGAGTG
corAORF1AUGfor	GACATTCGCCATGGACACACCCAGTAGATACTGG
Ptet-lacZ-38	TAGAGATTGACATCCCTATCAGTGATAGAGATACTGAGCACAATTGTGAGCGGATAACAATTTCAC
lacZ28-66rev	AACGCCAGGGTTTTCCCAGTCACGACGTTGTAAAACGAC
5'Ptet-mgtA	GATAGAGATTGACATCCCTATCAGTGATAGAGATACTGAGCACTAGATGCTACGAATATTATTGGA
3'mgtA-lacZ	GTAACGCCAGGGTTTTCCCAGTCACGACGTTGTAAAACGACAATGAGCCGGGTAAAAATTTCTT
5'lacI-P1mgtA	GCGAAGCGGCATGCATTTACGTTGACACCATCGAATGGCGCGTGCATTCAGCAATGGGTAAAGTC
mgtA-mutribo-for	GGATGCAATTATAATAATCGTTTTAACGTCCCTGCTCAGC
mgtA-mutribo-rev	TAAAACGATTATTATAATTGCATCCGGTAACGCCGTGAAAAG
F-corA-promoter	AAGAGGACATTCGCCTTG
R-corA-promoter	GGGATAGCCTTAGGAGTTG
F-corA1-UTR	GATACTGGCTCACTATCCTG
R-corA1-UTR	AAAATATCGGACAGTTCGGT
F-corA2-ORF	GAGTCCCGGTCATGCT
R-corA2-ORF	TGCATTTACAAGGGGTTG
F-corA3-ORF	CTGTGGCATTTACCATCC
R-corA3-ORF	CGTTACCGTCTACCATCG
F-corA4-ORF	AGATATCGGCTGGAAAGTTC
R-corA4-ORF	TGGAACAGGGATTCGTTATG
F-pLacUV5-corA	GGGCACCCCAGGCTTTACACTTTATGCTTCCGGCTCGTATAATGTGTGGGATCACTCTAAGAGGACATTCGCCTTGGAC

R-corA6codons	CCAGTTGAAATGCGCTCAGCATGAC
**Biotinylated oligonucleotides**	
corA probe	BioTEG-CGACTGGCTACGGGCACGCATACGATACAGACGAAAAGCG
5S probe	BioTEG-CTACCATCGGCGCTACGGCGTTTCACTTCTGAGTTCG

References

1. Lutz,R. and Bujard,H. (1997) Independent and tight regulation of transcriptional units in Escherichia coli via the LacR/O, the TetR/O and AraC/I1-I2 regulatory elements. *Nucleic acids research*, **25**, 1203–10.

2. Coornaert,A., Chiaruttini,C., Springer,M. and Guillier,M. (2013) Post-transcriptional control of the Escherichia coli PhoQ-PhoP two-component system by multiple sRNAs involves a novel pairing region of GcvB. *PLoS genetics*, **9**, e1003156.

3. Ghosh,T., Jahangirnejad,S., Chauvier,A., Stringer,A.M., Korepanov,A.P., Cote,J.-P., Wade,J.T. and Lafontaine,D.A. (2024) Direct and Indirect Control of Rho-Dependent Transcription Termination by the Escherichia coli lysC Riboswitch. *RNA*, 10.1261/rna.079779.123.

4. Guérout-Fleury,A.-M., Shazand,K., Frandsen,N. and Stragier,P. (1995) Antibiotic-resistance cassettes for Bacillus subtilis. *Gene*, **167**, 335–336.
